# Emotion regulation in bipolar disorder type-I: multivariate analysis of fMRI data

**DOI:** 10.1186/s40345-023-00292-w

**Published:** 2023-03-25

**Authors:** Fumika Kondo, Jocelyne C. Whitehead, Fernando Corbalán, Serge Beaulieu, Jorge L. Armony

**Affiliations:** 1 Douglas Mental Health University Institute, Verdun, QC Canada; 2 Integrated Program in Neuroscience, McGill University, Montreal, QC Canada; 3 Department of Psychiatry, McGill University, Montreal, QC Canada; 4 Department of Psychology, Université de Montréal, Montreal, QC Canada

**Keywords:** Bipolar disorder, fMRI, Emotion regulation, Multivariate pattern analysis, Reappraisal

## Abstract

**Background:**

Bipolar disorder type-I (BD-I) patients are known to show emotion regulation abnormalities. In a previous fMRI study using an explicit emotion regulation paradigm, we compared responses from 19 BD-I patients and 17 matched healthy controls (HC). A standard general linear model-based univariate analysis revealed that BD patients showed increased activations in inferior frontal gyrus when instructed to decrease their emotional response as elicited by neutral images. We implemented multivariate pattern recognition analyses on the same data to examine if we could classify conditions within-group as well as HC versus BD.

**Methods:**

We reanalyzed explicit emotion regulation data using a multivariate pattern recognition approach, as implemented in PRONTO software. The original experimental paradigm consisted of a full 2 × 2 factorial design, with valence (*Negative/Neutral*) and instruction (*Look/Decrease*) as within subject factors.

**Results:**

The multivariate models were able to accurately classify different task conditions when HC and BD were analyzed separately (63.24%–75.00%, *p* = 0.001–0.012). In addition, the models were able to correctly classify HC versus BD with significant accuracy in conditions where subjects were instructed to downregulate their felt emotion (59.60%–60.84%, *p* = 0.014–0.018). The results for HC versus BD classification demonstrated contributions from the salience network, several occipital and frontal regions, inferior parietal lobes, as well as other cortical regions, to achieve above-chance classifications.

**Conclusions:**

Our multivariate analysis successfully reproduced some of the main results obtained in the previous univariate analysis, confirming that these findings are not dependent on the analysis approach. In particular, both types of analyses suggest that there is a significant difference of neural patterns between conditions within each subject group. The multivariate approach also revealed that reappraisal conditions provide the most informative activity for differentiating HC versus BD, irrespective of emotional valence (negative or neutral). The current results illustrate the importance of investigating the cognitive control of emotion in BD. We also propose a set of candidate regions for further study of emotional control in BD.

**Supplementary Information:**

The online version contains supplementary material available at 10.1186/s40345-023-00292-w.

## Background

Bipolar disorder type I (BD-I) is characterized by fluctuating affective states, which can be attributed, at least in part, to impaired emotion regulation (ER). For instance, studies have identified the overuse of maladaptive ER strategies and the reduced use of adaptive cognitive techniques in BD-I (Dodd et al. 2019; Green et al. 2007, 2011; Wolkenstein et al. 2014). It has been suggested that the abnormal behaviour such as altered ER observed in BD patients is a result of the ineffective management of attentional and inhibitory resources (Picó-Pérez et al. 2017; Morris et al. 2012; Zhang et al. 2020). The functional and structural neuroimaging literature has identified the possible dysfunction of the inferior frontal gyrus (IFG), a region implicated in voluntary ER (Li et al. 2021; Ochsner and Gross 2005; Goldin et al. 2008), as a possible candidate brain region involved in this behavioural impairment. For instance, contrary to that of healthy controls (HC), BD patients’ lateral prefrontal cortex (PFC) grey matter volume did not correlate with measures of inhibitory mechanisms (Haldane et al. 2008) and structural alteration of PFC is linked to cognitive deficits in BD patients (Ajilore et al. 2015; Cipriani et al. 2017; Oertel-Knöchel et al. 2015). Also, although explicitly controlled ER activates IFG in HC (Braunstein et al. 2017), studies of ER in BD often report a hypoactive PFC, a hyperactive amygdala, and a disconnection between these two structures (Townsend and Altshuler 2012; Zhang et al. 2018; Kjærstad et al. 2021; Kanske et al. 2015; Morris et al. 2012). The hypoactivity of the PFC and the hyperactivity of the amygdala have been shown to enhance emotional response and impair regulation (Koenigsberg et al. 2010; McRae et al. 2010; Townsend et al. 2013). These findings support the characterization of BD as a condition that involves the PFC’s ineffective control over limbic regions, such as the amygdala, during ER.

In contrast to several aforementioned studies (Li et al. 2021; Townsend et al. 2013; Zhang et al. 2018; Kjærstad et al. 2021; Goldin et al. 2008; McRae et al. 2010; Kanske et al. 2015; Morris et al. 2012), our previous functional magnetic resonance imaging (fMRI) study (Corbalán et al. 2015) used a full-factorial ER paradigm (Ochsner et al. 2004), independently manipulating emotions and instructions. In doing so, we observed that BD-I patients could downregulate emotion when instructed, engaging largely the same regions as controls, particularly the left IFG. However, unlike HC, BD-I patients also activated these regions during the control condition, namely, when subjects were instructed to downregulate emotional responses associated with neutral images (for details, see Corbalán et al. 2015). These results reflected the patients’ responses when they were told to regulate their emotions, regardless of whether it was necessary (i.e. when presented with negatively valence stimuli) or not (i.e. in the presence of emotionally neutral images). This is consistent with the observation that BD patients show greater effort but are less successful in spontaneously regulating their emotions (Gruber et al. 2012). Additionally, while amygdala activity in HC decreased during ER, it was high in BD patients when exposed to negative stimuli regardless of instructions, which is similar to results found by Kanske et al. (2015). This suggests that BD patients demonstrated an exaggerated response to emotional stimuli, even when performing voluntary emotion downregulation.

Despite the important role of IFG, ER engages a network of frontal and parietal regions, likely acting in concert. For instance, past studies that used comparable ER paradigms observed the activation of parietal regions in addition to prefrontal areas during ER conditions (e.g. Ochsner et al. 2002; Townsend et al. 2013; Buhle et al. 2014; Diekhof et al. 2011). This was also demonstrated in our previous study, in which we observed inferior parietal lobe (IPL), middle frontal gyrus, cuneus, and lateral occipital complex activation, in addition to IFG, during ER conditions compared to control conditions (Corbalán et al. 2015). Beyond ventral frontal regions, response inhibition is accomplished through a distributed cortical network, including middle/inferior frontal gyri, limbic areas, insulae, and IPLs (Garavan et al. 1999). ER strategies also involve more general information processing systems, such as working memory and cognitive control, which recruit regions of the lateral frontal, inferior/superior parietal, and lateral occipital cortices (Kohn et al. 2014; Pozzi et al. 2021; Morawetz et al. 2017; Stephanou et al. 2016). Reappraisal has been shown to activate the lateral occipital cortex and IPL, including the supramarginal gyrus (Picó-Pérez et al. 2017; Ochsner et al. 2012; Buhle et al. 2014), regions associated with attentional selection and the storage of information for working memory (Culham and Kanwisher 2001). Notably, the parietal region acts as an association region that manages different functions, such as attention, working memory, and the guidance of actions, through activity modulation in the visual system. Additionally, the lateral PFC demonstrates strong connections to various secondary cortical association areas, including temporal, parietal, and occipital areas (Phillips et al. 2008; Moratti et al. 2011; Nguyen et al. 2019).

While a traditional univariate fMRI analysis can identify regions that significantly differ in activity between conditions and/or groups, it is less sensitive to small-magnitude differences in distributed networks (Lewis-Peacock and Norman 2013). In such a case, multivariate approaches, such as a multivariate pattern analysis (MVPA), may be better suited, through the computation of relative differences in the activation patterns and combining information from multiple voxels, leading to the detection of small pattern differences (Norman et al. 2006). Moreover, while a univariate General Linear Model (GLM) analysis reveals neural regions that are more active in response to one condition or group versus another, MVPA can detect differences in neural activation patterns across the brain and test whether different conditions or groups may be distinguished based on those distinct patterns. Thus, these analyses address two different yet complementary questions and can thus provide greater insight on brain functions, especially when investigating possible differences between psychiatric patients and HC.

Indeed, MVPA has been proposed as a potentially powerful tool for detecting certain mental illnesses (Schrouff et al. 2013a). In recent years, this approach has been applied to fMRI data in the study of psychiatric disorders, such as major depressive disorder (MDD), schizophrenia, and autism (Sundermann et al. 2014; Wolfers et al. 2015). The use of MVPA with fMRI data has been shown to be a promising tool for the early identification of psychiatric disorders, as models derived from previously acquired data can be used to predict the diagnostic outcome of a new sample (Mourão-Miranda et al. 2012). For example, Fu et al. (2008) demonstrated the potential of linear support-vector machines (SVM; Cortes and Vapnik 1995) for neuroimaging-based prediction of depression. In fact, machine learning has been applied in psychiatric fields using different imaging modalities including structural, functional, and diffusion MRI as a potential application to diagnose psychiatric disorders such as schizophrenia (see Steardo et al. 2020 for a review), MDD (Patel et al. 2016; Bhadra and Kumar 2022 for a review), and BD (Achalia et al. 2020; Li et al. 2020; Jan et al. 2021; Claude et al. 2020 for a review). MVPA has also been used for distinguishing between different psychiatric disorders (Sundermann et al. 2014), for example, between unipolar (major depressive disorder, MDD) and bipolar depression (Han et al. 2019; Grotegerd et al. 2014; Bürger et al. 2017; Rubin-Falcone et al. 2018). Moreover, it has also been applied to predict the risk of depression (Zhong et al. 2022) and BD (Hajek et al. 2015), as well as to identify clinical phenotypes of BD (Wu et al. 2017), brain volume changes in BD (Sartori et al. 2018), and to differentiate treatment response to electroconvulsive therapy in MDD (Redlich et al. 2016). Taken together, these studies confirm the potential of machine leaning techniques in clinical settings, particularly to discover novel biomarkers. However, many of these studies used structural MRI, diffusion-weighted imaging, resting-state or simple facial emotion processing fMRI tasks, and therefore the potential contributions of MVPA to a better understanding of the neural correlates of more complex cognitive and emotional processes, such as ER, remains less explored. This is particularly relevant, given that regulation of emotion involves, as mentioned above, the interaction between affective processing and executive functions, engaging a large network of cortical and subcortical regions. Because of this, multivariate approaches are ideally suited for investigating the neural correlates of such a process, and to characterize to what extent they may be affected in BD.

To address this issue, we conducted a new analysis of previously acquired fMRI data (Corbalán et al. 2015) using MVPA to determine whether this approach, when paired with an ER paradigm, may be used to distinguish between BD patients and HCs and determine whether the classification depends on the tasks performed by the subjects. Specifically, we assessed whether different experimental conditions could be accurately classified as a function of valence (negative vs. neutral) and instruction (passive viewing [PV] vs. downregulation of emotion [ER]) within each group and whether HC and BD individuals could be distinguished based on neural patterns of activity for each condition. We first conducted MVPA for four within-group classifications to assess whether the models differed in terms of performance and/or contributing regions between groups. We then conducted an MVPA with all subjects to determine if the model could classify individuals to their appropriate group using each condition of the ER paradigm and, if so, which brain regions contributed most to that classification. We hypothesized that the models would successfully classify different instruction (*Look*/*Decrease*) conditions, especially in the case of negative images. Moreover, we expected that the group classification model would perform better for the active ER conditions (i.e. downregulation) than for the passive viewing ones.

## Methods

The current study consisted in a novel analysis of the experiment described in Corbalán et al. (2015). A brief description of the participants, protocol and data acquisition is presented below. Further details can be found in the original publication.

### Participants

Nineteen euthymic BD-I patients (mean age: 41.0 ± 12.5 years) from the Bipolar Disorders Program at Douglas Mental Health Institute and seventeen healthy controls (mean age: 41.4 ± 13.3 years) participated in the study. The Faculty of Medicine Research Ethics Board of McGill University approved the study protocol. The study complied with the Helsinki Declaration of 1975, as revised in 2008. Written informed consent was provided by all participants. Diagnosis was carried out using the Structured Clinical Interview for Psychiatric Disorders of Axis I and II of the Diagnostic and Statistical Manual of the American Psychiatric Association (SCID-I/II DSM-IV; First et al., 1997a, b), as well as a documented manic phase. The Hamilton Depression Rating Scale (HAMD-29; Hamilton 1960), Montgomery–Asberg Depression Rating Scale (MADRS; Montgomery and Åsberg 1979), and the Young Mania Rating Scale (YMRS; Young et al. 1978) were used to confirm that patients were in a euthymic state at the time of the study. Exclusion criteria included the presence of any major Axis I psychiatric disorder for the HC group and the presence of an active co-morbid disorder associated to the diagnosis of bipolar-I disorder for the BD-I group. For both groups, exclusion criteria included a history of alcohol or drug abuse during the last year and use of benzodiazepines, as well as MR contraindications.

### Materials and procedure

**Fig. 1:**
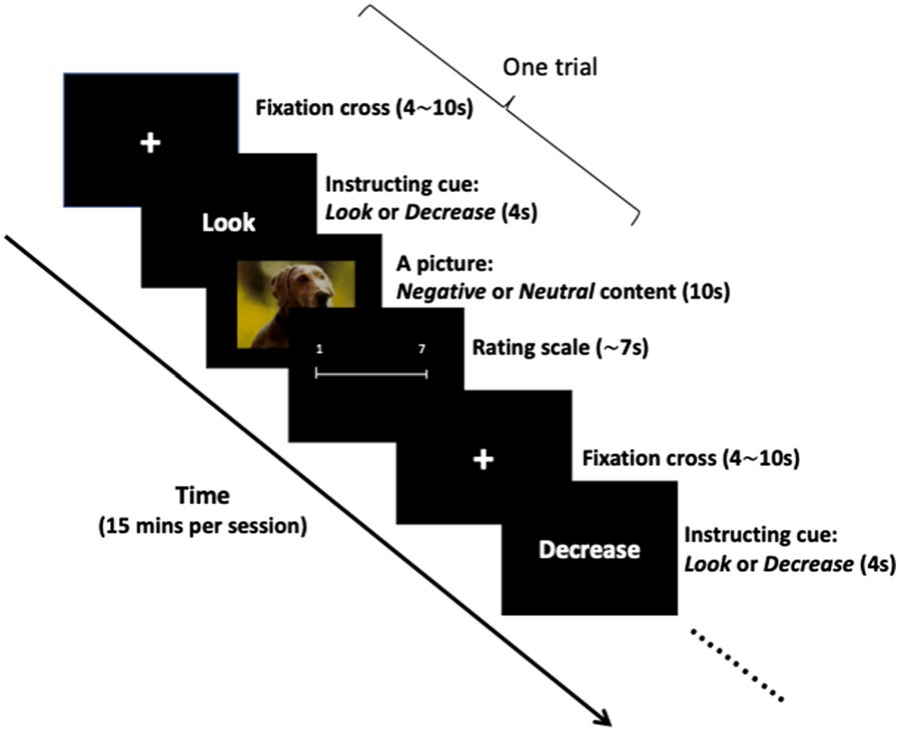
Schematic of the experimental paradigm. The experiment, in a full factorial, event-related design, consisted of 4 trial types, depending on the valence of the image (Negative/Neutral) and the instruction (Look/Decrease). Following each image, subjects rated how much negative affect they felt when viewing the picture. Trials were separated by a fixation cross lasting 4–10 s. For more details, see Methods and Corbalán et al. (2015)

The paradigm was adapted from Ochsner et al. (2004). Each trial started with a cue instructing participants to either look at the upcoming picture letting their emotions flow spontaneously (*Look* condition), or to decrease the negative affect induced by it through the previously practiced technique of reappraisal (*Decrease* condition). A picture selected from the International Affective Pictorial System (IAPS; Lang et al., 2008), showing either a negative or neutral scene, was then presented for 10 s, after which subjects rated their negative affect using a 7-point scale. Trials were separated by a fixation cross lasting between 4 and 10 s. Thus, the experiment was a full 2 × 2 factorial event-related design, with the valence (*Negative/Neutral*) of images and instructions (*Look*/*Decrease*) as the within-subject orthogonal factors, resulting in four event types (*Neutral Look, Neutral Decrease, Negative Look* and *Negative Decrease*). Each category contained sixteen images, presented in a pseudo-random order. The experiment was divided into two equivalent runs, each containing 32 trials and lasting about 15 min. A schematic of the experimental design is shown in Fig. 1.

## fMRI acquisition and analysis

### Acquisition

A total of 820 functional images (410 per run) were acquired using a 3 T Siemens Trio scanner with a 12-channel head coil at the Montreal Neurological Institute (MNI) in an interleaved fashion parallel to the anterior–posterior commissures, covering the whole-brain except the cerebellum (TE/TR: 30/2020 ms; flip angle: 90°; FOV: 256 × 256 mm^2^; matrix size: 64 × 64; voxel size = 4 × 4 × 4 mm^3^). A high-resolution T1-weighted scan (176 sagittal slices, voxel size: 1 mm^3^, TE/TR = 2.98/2300 ms) was obtained for spatial normalization of the functional images to a common template.

### Preprocessing and univariate analysis

Image preprocessing was done using SPM12 (v. 7771; Wellcome Trust Centre for Neuroimaging, UK), following the same approach as in our original analysis (Corbalán et al. 2015), but using a smaller smoothing kernel, as recommended for multivariate analyses (Gardumi et al. 2016). Briefly, the preprocessing steps included (1) slice timing correction (with the slice acquired at TR/2 as reference), (2) realignment to the first image of the first run, (3) co-registration to the participant’s T1, (4) normalization to the MNI template using the deformation field obtained in the T1 segmentation and normalization (final voxel size: 2 mm isotropic, bounding box: [− 78:78 − 112:76 − 70:85]), and (5) spatial smoothing with a 4 mm full-width half-maximum Gaussian kernel. Mean and variance images were constructed for each subject and run after every preprocessing step and inspected for possible artifacts. Based on the analysis of the six movement parameters obtained in the realignment step and the voxelwise inter-volume signal differences (see Additional file 1), there were no significant differences in motion during scanning between groups (Additional file 1: Table S1).

The univariate data analysis was also performed in SPM12, in the context of the GLM. Four event types representing the experimental categories (*Neutral Look*, *Neutral Decrease*, *Negative Look*, *Negative Decrease*) were used to build first-level design matrices for each run and participant. To account for possible temporal differences in evoked responses during the 10 s presentation of the images (Ochsner et al. 2002, 2004), the first (0–5 s) and second half (5–10 s) of the stimulus presentation were modeled separately by two adjacent 5-s boxcars convolved with the canonical hemodynamic response function (for more details, see Penny et al. 2007). That is, the beginning of the first boxcar coincided with the onset of the image, whereas the second started 5 s later. In addition, the six movement parameters from the realignment process were included as covariates. For the univariate second-level analysis, the parameter estimates (*betas*) for each subject and run were entered in a mixed-effects linear mixed model with image time (Early/Late), instruction (*Look/Decrease*) and valence (*Negative/Neutral*) as the within-subject factors and group (BD/HC) as the between-subjects factor, with *Run* as a factor of no interest (and thus averaged when computing contrasts). Statistical significance was determined using a voxel threshold of *p* = 0.001, with a cluster-based familywise error rate (FWE) correction for multiple comparisons of *p* < 0.05 (*k* = 248) as implemented in AFNI’s 3dClustSim (AFNI version 19.0.17).

### MVPA analysis

*Beta* images specific to each condition, run, and subject obtained from the univariate GLM were used in the MVPA as implemented in PRoNTo v2.1.3 (Pattern Recognition Neuroimaging Toolbox; Schrouff et al. 2013b, 2016). A kernel classifier was trained to identify voxel activation patterns in *beta* images using a binary linear SVM (Cortes and Vapnik 1995). The SVM calculates a hyperplane, which optimally separates the two conditions of interest (Grotegerd et al. 2013) and identifies the boundary that maximizes the overall classification accuracy of each new sample (Costafreda et al. 2011). Classifiers were trained to detect voxel activation patterns of the *beta* images using a leave-one-subject-out (LOSO) cross-validation, in which the model is trained using all subjects minus one. Then, the remaining subject is used to test the accuracy level of the prediction. During training, the classifier assigns a weight to each voxel, and then uses these weights to calculate the summed weight of activity during testing. This information is used to determine whether the pattern of activity of each new subject falls on the predicted side of the decision boundary or not (Lewis-Peacock and Norman 2013). From this model binary classification analyses were conducted across two conditions or subject groups at a time *(beta* images from both runs were combined). Model performance was assessed in terms of the balanced accuracy as well as the *sensitivity* and *specificity*, i.e. the accuracies representative of each condition or group, with *Decrease* and BD assigned as the target (“positive”) categories, respectively. The balanced accuracy accounts for the number of samples in each class and in doing so, distributes equal weight to the classes. Significance of the classifier’s performance was set at *p* = 0.05, calculated through random permutations of the training labels, in which the classification model is retrained 1000 times. A mask of all the regions, except the cerebellum, from the Automated Anatomical Labeling atlas v1 (Tzourio‐Mazoyer et al. 2002), as implemented in the WFU Pickatlas Tool v2.4 (Maldjian et al. 2003, 2004) was created in MNI space; only the voxels inside the mask (size: 159,841 voxels) were included for pattern recognition. In order to make comparisons between different classifications in terms of its prediction accuracy, Cohen’s Kappa values were calculated for each classification (Forbes 1995). The Kappa calculation takes into account the estimated and observed proportions, in which larger Kappa values indicate better predictions and less error. In addition, a single binomial contrast was calculated for the comparison between proportions of two different confusion matrices (Rodríguez-Avi et al. 2018). The *p*-values of binomial analyses indicate if the prediction accuracy of one classification was significantly different from that of another.

### Weight map

Results from the classification analysis are typically presented as a weight map, which illustrate areas that present the greatest contribution to discriminating between the different groups/conditions. Importantly, a high weight value of a specific voxel indicates a strong contribution to the discrimination boundary but does not directly mean greater activity in one group versus another within the specified voxel (Mourão-Miranda et al. 2012). To identify the regions that most contributed to the classification in each model, clusters corresponding to the top 5% of the positive and negative weights, collectively, having a minimum cluster size of 5 voxels, were extracted. The *p*-value associated with the weight for each voxel was obtained from the permutation test (see previous section). Threshold of significance was set at *p* = 0.01.

## Results

### Behavioral and univariate results

As described in more detail in Corbalán et al. (2015), subjective ratings for the intensity of the negative emotional experience revealed that both groups considered the *Negative Decrease* condition significantly less intense than the *Negative Look* condition, with no significant differences between groups. In terms of the fMRI analysis, results obtained in the analysis conducted here were qualitatively similar, in terms of the brain regions significantly activated in the different contrasts of interest, with those described in the original paper (Corbalán et al. 2015), as shown in Additional file 1: Fig. S1 and Additional file 1: Table S2. Briefly, a conjunction analysis (HC and BD) of the contrast *Negative Decrease* minus *Negative Look* confirmed that both groups showed comparable activations in areas previously associated with emotion regulation, including the left IFG, when asked to downregulate their emotional reaction to negative pictures, with no significant differences between groups. On the other hand, in the case of the *Neutral Decrease* minus *Neutral Look* contrast, a significant group difference was observed in the left IFG, driven by a significant activation for this contrast only in the BD group.

### Multivariate analysis

Classifications were first conducted within group, in which the model was trained to classify the two conditions: *Neutral Decrease* versus *Neutral Look,* or *Negative Decrease* versus *Negative Look*. Classifier model performance reported in Tables 1 and 2 is expressed in terms of classification accuracy and Kappa scores. The multivariate approach produced statistically significant accurate classifications across task conditions (*Decrease* and *Look*) when HC and BD were analyzed separately, for both *Negative* (HC: 64.7%; BD: 71.1%) and *Neutral* (HC: 63.2%; BD: 75.0%) conditions. That is, above-chance accuracies were obtained in each within-group classification, with slightly higher levels of both sensitivity (i.e. correctly classified *Decrease* trials) and specificity (i.e. correctly classified *Look* trials) for the BD than the HC group (Table 1). Likewise, Kappa values were higher in BD compared to HC in each classification; *Neutral Decrease* vs. *Neutral Look* (BD: 0.50, HC: 0.26)*,* and *Negative Decrease* vs. *Negative Look* (BD: 0.42, HC: 0.29) (Table 2). Finally, a single binomial contrast suggested that confusion matrices for the classification *Neutral Decrease* versus *Neutral Look* were significantly different between HC and BD (*p* = 0.03), while confusion matrices for *Negative Decrease* versus *Negative Look* did not significantly differ between groups (*p* = 0.25) (Table 3A).

**Table 1:** Classification accuracy for each model

Classification	Balanced accuracy (%)	Sensitivity	Specificity
HC			
Negative decrease versus negative look	64.71 (*p* = 0.005)	63.24	66.18
Neutral decrease versus neutral look	63.24 (*p* = 0.012)	67.65	58.82
BD			
Negative decrease versus negative look	71.05 (*p* = 0.001)	69.74	72.37
Neutral decrease versus neutral look	75.00 (*p* = 0.001)	75.00	75.00
HC versus BD			
Negative decrease	60.84 (*p* = 0.014)	65.79	55.88
Neutral decrease	59.60 (*p* = 0.018)	61.84	57.35
Negative look	48.14 (*p* = 0.511)	56.58	39.71
Neutral look	42.41 (*p* = 0.861)	53.95	30.88

Abbreviations are as follows: HC = healthy controls; BD = bipolar disorder patients. *Sensitivity* refers to accurate classification of *Decrease* trials or BD subjects, whereas *Specificity* indicates the percent of accurately classified for *Look* trials or HC subjects

**Table 2:** Kappa values of each classification

Classification	Kappa values
(HC) N_G_D versus N_G_L	0.29
(HC) N_E_D versus N_E_L	0.26
(BD) N_G_D versus N_G_L	0.42
(BD) N_E_D versus N_E_L	0.50
(N_G_D) HC versus BD	0.22
(N_E_D) HC versus BD	0.19
(N_G_L) HC versus BD	− 0.04
(N_E_L) HC versus BD	− 0.15

Larger values indicate better predictions. Abbreviations are as follows: HC = healthy controls; BD = bipolar disorder patients; N_E_D = *Neutral Decrease*; N_E_L = *Neutral Look;* N_G_D = *Negative Decreas*e; N_G_L = *Negative Look*

**Table 3:** Binomial contrast between confusion matrices

A. Group comparison for *Decrease* versus *Look* classification		
	Z-score	*p*-value
(N_E_D vs. N_E_L) HC only	–2.164	0.0305
**Versus**		
(N_E_D vs. N_E_L) BD only		
(N_G_D vs. N_G_L) HC only	–1.153	0.2488
**Versus**		
(N_G_D vs. N_G_L) BD only		

B. Condition comparison for HC vs. BD classification		
	Z-score	*p*-value
(HC vs. BD) N_G_D	2.131	0.0331
**Versus**		
(HC vs. BD) N_G_L		
(HC vs. BD) N_G_D	0.241	0.8096
**Versus**		
(HC vs. BD) N_E_D		
(HC vs. BD) N_E_D	2.83	0.0047
**Versus**		
(HC vs. BD) N_E_L		
(HC vs. BD) N_G_L	0.946	0.3441
**Versus**		
(HC vs. BD) N_E_L		

Subsequently, the model was trained to distinguish HC and BD individuals for each of the four different conditions. The classification of HC and BD was above chance for *Negative* and *Neutral Decrease* conditions (60.8% and 59.6%), but not for *Negative* or *Neutral Look*, which yielded an accuracy of only 48.1% and 42.4%, respectively. For the HC versus BD classifications, average value for sensitivity (true positives for BD) was 59.5% (significant models alone = 63.8%) and for specificity (true positives for HC) was 46.0% (significant models alone = 56.6%), suggesting the classifier was better at discriminating the BD subjects, while HC were more often misclassified as BD. Kappa values revealed that *Negative Decrease* condition produced the most successful prediction (Kappa = 0.22), followed by *Neutral Decrease* (Kappa = 0.19), indicating that, consistent with the prediction accuracy rates described above, the two groups could be better classified in the *Decrease* conditions, compared to *Look* conditions (Table 2). This was confirmed by a single binomial contrast revealing that HC and BD subjects were significantly more accurately classified for *Neutral Decrease* condition compared to *Neutral Look* condition (*p* = 0.005), and for *Negative Decrease* than *Negative Look* (*p* = 0.03) (Table 3B). The ranking of balanced accuracy for the significant classifications is shown in Table 4.

**Table 4:** Ranking of balanced accuracy

	Classification	Balanced Accuracy (%)
1	(BD) N_E_D versus N_E_L	75.00 (*p* = 0.001)
2	(BD) N_G_D versus N_G_L	71.05 (*p* = 0.001)
3	(HC) N_G_D versus N_G_L	64.71 (*p* = 0.005)
4	(HC) N_E_D versus N_E_L	63.24 (*p* = 0.012)
5	(N_G_D) HC versus BD	60.84 (*p* = 0.014)
6	(N_E_D) HC versus BD	59.60 (*p* = 0.018)

The ranking includes significant classifications only. Abbreviations are as follows: HC = healthy controls; BD = bipolar disorder patients; N_E_D = *Neutral Decrease*; N_E_L = *Neutral Look;* N_G_D = *Negative Decreas*e; N_G_L = *Negative Look*

### Brain regions

**Fig. 2:**
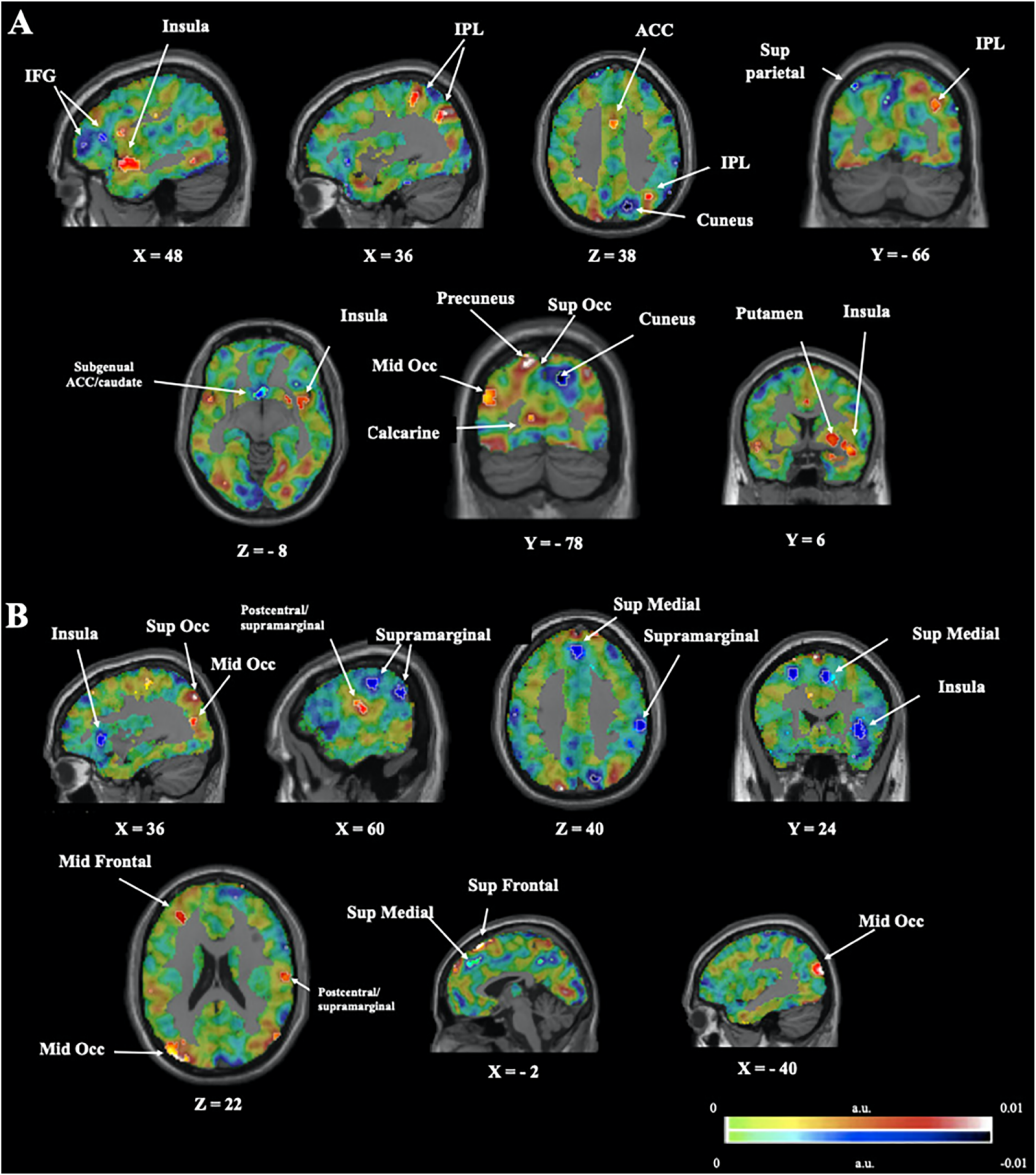
Weight map of positive and negative voxel-wise weight contribution in the classification of HC versus BD. A:* Negative Decrease* HC versus BD. B:* Neutral Decrease* HC versus BD. Highlighted region depicts contribution of the top 5% positive (red) and negative (blue) weights, with a minimum extent of 5 voxels. Abbreviations are as follows: a.u. = arbitrary units; Mid Occ = middle occipital cortex; Sup Occ = superior occipital cortex; IFG = inferior frontal gyrus; IPL = inferior parietal lobe; ACC = anterior cingulate cortex; Mid frontal; middle frontal; Sup Medial = superior medial; Sup Frontal = superior frontal. Images are shown in neurological convention (i.e. right hemisphere is on the right).

Weight maps showing the top 5% voxels that contributed most to the classification (see Methods) are shown in Fig. 2 for the between-group classifications and Additional file 1: Fig. S2  for the within-group classifications. Discrimination between conditions engaged, in both groups, a number of regions spanning across the entire brain, several of them within the frontal lobes, including those considered part of the Salience Network (SN), such as ACC and Insula, as well as occipital visual-related, areas. No obvious difference in the weight patterns appeared to exist between image valence (*Negative*/*Neutral*) or group (HC/BD), suggesting that in all cases, discrimination of conditions as a function of Instruction (*Look*/*Decrease*) engaged a large distributed network of cortical and subcortical regions.

For the group classification of HC versus BD using *Negative Decrease* (Fig. 2A; Additional file 1: Table S3A)*,* the clusters corresponding to the top 5% of voxels were located in right insula, right putamen, also located broadly in the visual processing regions (left middle occipital, right cuneus, left superior occipital, left calcarine), cognitive control regions (left subgenual ACC/caudate, bilateral IPL, superior parietal, right IFG). Regions that contributed the most to discriminating the HC versus BD for *Neutral Decrease* condition (Fig. 2B; Additional file 1: Table S3B) included visual processing regions (left middle/superior occipital), cognitive control regions (right superior medial, left middle/superior frontal, right supramarginal gyrus), and right insula. Hence, for the significant classifications of HC versus BD, the weight maps of the most discriminative voxels were located primarily in the occipital regions, as well as prefrontal areas, insula, also parietal regions such as IPL/supramarginal gyrus.

## Discussion

The current study implemented a multivariate classification analysis in an emotion regulation fMRI task to identify differences in whole-brain patterns across conditions (regulations vs. passive viewing) and subject populations (HC vs. BD patients). The results suggest that reappraisal conditions provide the most informative activity for differentiating HC versus BD, irrespective of emotional valence (*Negative* or *Neutral*). Nevertheless, when no instructions were given, the patterns across groups were relatively similar at the whole-brain pattern level, which may be due to lower cognitive demands required, compared to higher cognitive demands during the *Decrease* conditions (i.e. regulation). It has been reported that when tasks only require simple emotion reactivity by bottom-up processing, in this case passive viewing, euthymic BD patients tend to exhibit similar activation as HC (Townsend et al. 2013; Foland-Ross et al. 2012; Hassel et al. 2009), where both groups demonstrate amygdala activation and no difference in frontolimbic functioning is present.

Interestingly, contrary to the significant classifications by the current multivariate model, neither *Decrease* condition (N_G_D, N_E_D) demonstrated group differences behaviourally in terms of negative emotional experience (Corbalán et al. 2015). Moreover, there was no group (HC vs. BD) difference for the univariate N_G_D–N_G_L contrast, but the MVPA demonstrated the highest group classification accuracy for N_G_D, while there was below-chance level classification for N_G_L. As BD performed as well as HC behaviourally, distinct neural patterns between HC vs. BD in N_G_D may represent a compensatory processing in BD to achieve the same results as HC while performing emotion regulation, which was not observed in the univariate analysis. This process likely occurs to compensate for interference from autonomic emotional reaction (Strakowski et al. 2005; Picó-Pérez et al. 2017) to maintain emotion regulation performance, which can be seen in the high negative weight of subgenual ACC and IFG in the group classification of N_G_D, as both regions are involved in modulation of amygdala processing toward emotional stimuli in patients with mood disorders (Campbell-Sills et al. 2011; Drevets et al. 2008). The development of such compensatory mechanisms may be the result of repeated trial-and-error experiences with this disorder, when individuals with BD-I encounter situations requiring emotion regulation (Kollmann et al. 2017).

As for the N_E_D condition, univariate analysis showed significant group difference in the contrast N_E_D–N_E_L (see Additional file 1: Table S2 and Additional file 1: Fig. S1), and the current MVPA demonstrated a significant group classification accuracy for N_E_D, but not for N_E_L. These results confirm that the N_E_D condition itself has group differences in underlying neural patterns, and not in N_E_L. Taken together with univariate results, it indicates an existence of heightened neural processing in BD after situation-incongruent (i.e. unnecessary) emotion regulation instruction. Heightened neural processing during N_E_D in BD can be also presumed from within-group classifications of N_E_D vs. N_E_L where the classification accuracy of BD was significantly higher than that of HC (Tables 2, 3A). This confirms the existence of distinct neural processing that emotion regulation instructions produce in HC vs. BD, even with neutral images. We speculate that this discrepancy may reflect euthymic BD patients’ elevated impulsivity (Ramírez-Martín et al. 2020; Mason et al. 2014) and impaired decision-making towards environmental stimuli (Bhatia et al. 2018; Adida et al. 2011; Martino et al. 2011); in our case, a combination of the neutral images and emotion regulation instruction.

Contrary to past MVPA studies involving patients with BD (Claude et al. 2020; Doan et al. 2017; Costafreda et al. 2011), in the current study the model yielded fewer misclassifications of BD patients compared to the HC group, especially in the case of the N_G_D condition (Table 1). It is known that differences in classification accuracy is determined by group differences in variance parameter (Koch et al. 2015). Although emotion regulation has high individual differences (Dickie and Armony 2008), there should be a reduction of individual differences (Drucaroff et al. 2020; Calhoun et al. 2008) in both groups because MVPA is robust towards between-subject variability in mean activation (Weaverdyck et al. 2020; Davis et al. 2014; Kryklywy et al. 2018). Since there is a clinical heterogeneity in BD patients, higher accuracy of BD may be due to an overall smaller variance in HC, which caused classification models to mis-assign any outliers in the HC group into the BD group. Our results suggest that tasks with explicit ER may be more discriminative for BD than for HC.

### Brain networks

As shown in the Tables (Additional file 1: Table S3) and figures (Additional file 1: Fig. S2), and mentioned in the Results, accurate discrimination between conditions relied on a large number of cortical and subcortical regions. This is consistent with the notion that emotion regulation involves several cognitive and affective processes, such as emotional evaluation, attention and cognitive control (Gross 2015; Thompson et al. 2019; Pruessner et al. 2020), which are effected by different, yet possibly overlapping, regions and networks, such as the Salience and Fronto-Parietal/Central Executive ones (Pan et al. 2018; Li et al. 2021; Morawetz et al. 2020; Phan et al. 2005; Kalisch 2009; Menon and Uddin 2010; Peters et al. 2016). These findings also highlight the relevance of using multivariate approaches to study this process. Indeed, whereas univariate analysis typically reveals a few regions where the difference in activity between conditions is maximal (Additional file 1: Table S2 and Additional file 1: Fig. S1), MVPA can capture the distributed pattern of activity, including voxels with relatively small signal, actually involved in the different processes (Jimura and Poldrack 2012; Norman et al. 2006; Davis et al. 2014).

The observed high contribution of regions in the SN, such as the Insula, and occipital visual areas in HC vs. BD classification of N_G_D (Additional file 1: Table S3A) likely to reflect a high contribution of these regions in classifying N_G_D vs. N_G_L within HC (Additional file 1: Table S3C). Similarly, high contribution of the middle occipital cortex and regions in fronto-parietal network (FPN) including the postcentral/supramarginal and middle/superior frontal in HC vs. BD classification of N_E_D (Additional file 1: Table S3B) is likely reflecting a high contribution of these regions in classifying N_E_D versus N_E_L within HC (Additional file 1: Table S3D). Hence, based on top weighted regions observed in the category and group classifications, it seems that a neural pattern of the primary visual region is distinct following emotion regulation instruction vs. no instruction in HC, and also distinct from that of BD after emotion regulation instruction. This may indicate distinct visual processing in BD compared to HC after emotion regulation instruction, which is consistent with the notion of altered visual information processing in euthymic BD (Maekawa et al. 2013; Morsel et al. 2018; Yeap et al. 2009). Indeed, it has been suggested that the disturbances of the visual system could be used as a diagnostic indicator for psychotic diseases that display generalized attentional deficit (Bellani et al. 2020). It would be meaningful to examine the relationship between visual processing and cognitive/neuropsychological functions of BD patients to further understand this population’s potential sensory impairments. Moreover, distinct processing of the insula during N_G_D may indicate that this region, known for its role in salience detection (Uddin 2015; Menon and Uddin 2010) and assigning salience in response to contextual demands (Jiang et al. 2015; Power and Petersen 2013), is important in executing efficient emotion regulation when it is necessary. Insula was reported to be a key part of the cognitive neural system during the emotion regulation process using the reappraisal (Picó-Pérez et al. 2019; Steward et al. 2016). In particular, increased association of insula with IFG indicates a better regulation effect of reappraisal (Li et al. 2021). The insula is also known to work together with frontoparietal regions and form a frontoparietal control network (Cauda et al. 2012; Vincent et al. 2008; Molnar-Szakacs and Uddin 2022). This network controls information processing under variety of strategies to enhance the regulation of emotions (Li et al. 2021; Morawetz et al. 2017; Kanske et al. 2011; Goldin et al. 2008; Moodie et al. 2020; Dörfel et al. 2014). High contribution of the SN and visual regions in group classification of N_G_D may imply distinct processing in BD when focusing on the emotionally salient components of the image after the regulation instruction because directing attention to the appropriate target is the first step in the emotion regulation process (Dickstein and Leibenluft 2006). Furthermore, distinct processing of FPN regions (postcentral/supramarginal, middle/superior frontal) during N_E_D may suggest that this network, known for its role in executing goal-oriented, cognitively demanding task (Uddin et al. 2019; Seeley et al. 2007; Vincent et al. 2008; Dosenbach et al. 2008), is contributing to accurately judging if emotion regulation is necessary. High contribution of regions relating to the salience and frontoparietal attentional networks are consistent with reported deficits in these regions in BD during regulation of cognitive-affective integration task (Ellard et al. 2018; Rai et al. 2021).

### Comparison with univariate analysis

Although the BD group alone demonstrated significant activity in the univariate analysis for the contrast regulation versus viewing with neutral images (N_E_D vs. N_E_L) (Additional file 1: Table S2), the classification model significantly classified these two conditions within the HC group as well. This may reflect the characteristics of MVPA to detect subtle differences of mean-responses in a whole brain network (Coutanche 2013; Kragel et al. 2012) due to the content of the stimuli or the task instructions (Todd et al. 2013). Therefore, in our case, subtle response differences following different task instructions could have been detected by the model in HC as well. That said, this classification (regulation vs. viewing of neutral images) within HC produced the lowest accuracy among all within-group classifications and was significantly lower than the same classification of BD (see Tables 3A, 4).

Surprisingly, top contributing regions reported from within-group classifications differed from regions of activity observed in univariate analyses, for example the IFG. In HC, classifying regulation versus viewing conditions with negative images showed the ACC and insula, both of which belong to the SN (Seeley 2019). Here, other effects, such as condition-related attentional processing and perception changes under different task conditions may have been detected by MVPA (Jimura and Poldrack 2012; Kragel et al. 2012; Coutanche 2013; Davis and Poldrack 2013; Ritchie et al. 2019; Todd et al. 2013) rather than emotion regulation or passive viewing processing. Similarly, although the univariate analysis revealed that BD patients did not exhibit decreased amygdala activation during N_G_D condition (Additional file 1: Fig. S3), as observed in HC, this region did not notably contribute to the group classification. The lack of amygdala contribution is unlikely due to the use of multivariate analysis, as our previous work observed such activity when classifying emotion-related conditions (Whitehead and Armony 2019). Rather, it could be explained by the fact that emotion regulation paradigms require engagement of more dispersed regions and broader neural networks than simple emotion-processing paradigms (Braunstein et al. 2017; Townsend and Altshuler 2012).

### Limitations and future directions

A limitation of the current study is that medication use was not included in the analysis, although our univariate analysis did not show significant differences with and without the index of medication use as a covariate. Likewise, although some potential confounding factors, such as age, IQ and number of years of formal education were matched between two subject groups, and therefore unlikely to have driven the results, we cannot completely rule out their having an effect on the classification accuracy. Another important limitation is our relatively small sample size. Because of this, only limited conclusions can be drawn, and the current results must be replicated and validated on large samples in future studies. Moreover, as our study only investigated one form of cognitive control and one type of emotion regulation, future studies would benefit from including non-emotional cognitive control or other forms of emotion regulation (e.g. suppression) to explore the generalizability of the current findings. In addition, as BD patients have difficulty regulating positive stimuli (Gruber 2011a, 2011b), it would be interesting to include such stimuli in future experiments.

## Conclusion

The multivariate approach provided significant contributions to our understanding of dissimilar brain activation pattern in BD compared to HC following emotion regulation instructions, adding to results obtained in the previous univariate analysis. Specifically, although univariate analysis reported that neural processing in BD is different from HC only when there is unnecessary emotion regulation instruction (Corbalán et al. 2015), the current MVPA indicates that distinct neural processing exists even when emotion regulation is context-congruent. This implies that emotion regulation instruction itself causes a distinct processing mechanism between groups regardless of the valence. Comparing these findings with behavioural and univariate results, it can be assumed that there is a compensatory mechanism in BD to achieve successful emotion regulation. Accordingly, we provided a set of candidate regions for further study. Based on the top contributing regions detected by the classifier, that is insula, visual attention regions, IPL, and IFG, it can be assumed that BD patients exhibit a distinct recruitment of attentional and cognitive control network from HC. Moreover, the neural pattern of the middle occipital cortex was distinct between groups in both emotion regulation conditions, indicating this region may contain fundamental information on how cognitive control instructions affect individuals with BD. Similarly, insula and FPN recruitment appear critical for effective use of the external information, specifically efficient emotion regulation when considering cognitive load. This may be a candidate trait marker of unique emotion regulation processing in BD and may reflect a circuit-level etiology of BD. These conclusions could not be drawn from the behavioral or univariate analyses alone, and therefore support the importance of incorporating multivariate methods when studying psychiatric patients. The current findings also suggest that future research on BD should place importance on the cognitive control of emotion versus emotion processing, as BD distinct characteristics appear to depend on the former.

## Supplementary Information

**Additional file 1.** **Table S1.** Movement-related parameters during scanning. **Table S2.** Coordinates and brain regions from the univariate analysis. **Table S3.** Clusters corresponding to the top 5% of the weight contribution in significant classifications. **Table S4.** Top 10 regions contributing to the significant group classifications based on the combined cluster size**. Figure S1.** Statistical parametric maps of the contrast *Negative Decrease—Negative Look* and *Neutral Decrease—Neutral Look*. **Figure S2.** Weight map of positive and negative voxel-wise weight contribution in the within-group classifications. **Figure S3.** Amygdala activation for the contrast *Negative Look—Negative Decrease* for the HC group (*x* = 20, *y* = − 4, *z* = − 18).

## Data Availability

The datasets used and/or analysed during the current study are available from the corresponding author on reasonable request.

## Abbreviations

BD: Bipolar disorder patients
HC: Healthy controls
MDD: Major depressive disorder
GLM: General linear model
MVPA: Multivariate pattern analysis
SVM: Support vector machine
ER: Emotion regulation
PV: Passive viewing
PFC: Prefrontal cortex
IFG: Inferior frontal gyrus
IPL: Inferior parietal lobe
ACC: Anterior cingulate cortex
FPN: Fronto-parietal network
SN: Salience network
N_*G*_D: Negative decrease
N_*E*_D: Neutral decrease
N_*G*_L: Negative look
N_*E*_L: Neutral look
