# Supplementary material for: Emotion regulation in bipolar disorder type-I: multivariate analysis of fMRI data
Source: Int J Bipolar Disord. 2023 Mar 25;11:12. doi: 10.1186/s40345-023-00292-w (PMC10039967; doi:10.1186/s40345-023-00292-w)
Supplement: Supplementary file 1 — Additional file 1. Table S1. Movement-related parameters during scanning. Table S2. Coordinates and brain regions from the univariate analysis. Table S3. Clusters corresponding to the top 5% of the weight contribution in significant classifications. Table S4. Top 10 regions contributing to the significant group classifications based on the combined cluster size. Figure S1. Statistical parametric maps of the contrast Negative Decrease—Negative Look and Neutral Decrease—Neutral Look. Figure S2. Weight map of positive and negative voxel-wise weight contribution in the within-group classifications. Figure S3. Amygdala activation for the contrast Negative Look—Negative Decrease for the HC group (x = 20, y = − 4, z = − 18). [file 40345_2023_292_MOESM1_ESM.docx]

**SUPPLEMENTARY MATERIAL**

**Analysis of head movement during scanning**

We computed the root mean square (RMS) and maximum absolute value of the derivative (i.e., inter-volume differences) of each of the six parameters obtained in the realignment procedure, for each subject. Additionally, we calculated the actual inter-volume displacement of each voxel in the brain for each subject and computed the RMS and maximum value across scans and voxels. Finally, we computed the mean normalized voxelwise inter-volume signal difference (DVARS; Smyser et al., 2010, Power et al., 2012) for each subject. The values obtained, shown in Table S1, were submitted to independent-sample t-tests. No significant differences between groups was observed for any of these parameters.

References

Power JD, Barnes KA, Snyder AZ, Schlaggar BL, Petersen SE (2012). *Neuroimage* 59: 2142-2154.

Smyser CD, Inder TE, Shimony JS, Hill JE, Degnan AJ, Snyder AZ, Neil JJ. (2010). *Cerebral Cortex* 20: 2852-2862

Supplementary Tables

**Table S1: Movement-related parameters during scanning**

| Parameter |  | HC |  | BD |  |  |
| --- | --- | --- | --- | --- | --- | --- |
|  |  | mean | SD | mean | SD | p-value |
| x | RMS | 0.018 | 0.009 | 0.027 | 0.031 | 0.20 |
|  | max | 0.162 | 0.113 | 0.369 | 0.834 | 0.30 |
| y | RMS | 0.114 | 0.041 | 0.127 | 0.049 | 0.41 |
|  | max | 0.529 | 0.281 | 0.604 | 0.214 | 0.38 |
| z | RMS | 0.086 | 0.050 | 0.104 | 0.056 | 0.31 |
|  | max | 0.579 | 0.343 | 0.655 | 0.364 | 0.53 |
| pitch | RMS | 0.001 | 0.001 | 0.001 | 0.001 | 0.85 |
|  | max | 0.014 | 0.011 | 0.011 | 0.010 | 0.28 |
| yaw | RMS | 0.000 | 0.000 | 0.001 | 0.000 | 0.26 |
|  | max | 0.004 | 0.003 | 0.007 | 0.010 | 0.27 |
| roll | RMS | 0.001 | 0.000 | 0.001 | 0.001 | 0.43 |
|  | max | 0.005 | 0.005 | 0.008 | 0.011 | 0.29 |
| displacement | RMS | 0.126 | 0.053 | 0.156 | 0.066 | 0.15 |
|  | max | 1.774 | 1.300 | 1.892 | 1.864 | 0.83 |
| DVARS | mean | 0.037 | 0.006 | 0.040 | 0.009 | 0.17 |

**Table S2: Coordinates and brain regions from the univariate analysis**

|  | **MNI coordinates** | | |  |
| --- | --- | --- | --- | --- |
| **Region** | **x** | **y** | **z** | **z score** |
| **HC: N_G_D - N_G_L** |  |  |  |  |
| L IFG | -48 | 30 | -12 | 4.7 |
| L Mid temporal | -48 | -40 | 0 | 4.22 |
| L SMA | -4 | 10 | 64 | 4.13 |
| **BD: N_G_D - N_G_L** |  |  |  |  |
| L IFG | -50 | 26 | -8 | 4.95 |
| R DLPFC | 42 | 8 | 36 | 4.29 |
| L Lingual | -8 | -78 | 0 | 5.48 |
| L SMA | -8 | 6 | 68 | 5.04 |
| L Precentral | -46 | 2 | 46 | 4.98 |
| L Mid temporal | -62 | -56 | 16 | 4.59 |
| R Sup temporal | 44 | -42 | 4 | 4.56 |
| L Mid OCC | -46 | -84 | 4 | 4.07 |
| L Parahippocampal | -12 | -28 | -20 | 3.9 |
| **HC and BD: N_G_D-N_G_L (conjunction)** | | | | |
| L IFG | -46 | 28 | -10 | 4.49 |
| **BD: N_E_D - N_E_L** |  |  |  |  |
| L IPL | -48 | -62 | 44 | 5.87 |
| R IPL | 58 | -58 | 42 | 5.49 |
| L IFG | -50 | 36 | -2 | 4.95 |
| R DLPFC | 36 | 16 | 42 | 4.51 |
| R IFG | 44 | 48 | -8 | 4.5 |

MNI, Montreal Neurological Institute; HC, healthy controls; BD, bipolar disorder; IFG; inferior frontal gyrus; SMA, supplementary motor area; IPL, inferior parietal lobe; DLPFC, dorsolateral prefrontal cortex; OCC, occipital cortex; L, left; R, right.

**Table S3:** **Clusters corresponding to the top 5% of the weight contribution in significant classifications.** Peak coordinates of cluster, weights, and cluster size reported for each anatomical location, sorted in terms of cluster size. Weight indicates the average contribution of the ROIs to the classification. Only clusters equal to or greater than 5 voxels (40 mm^3^) are reported. Abbreviations are as follows: R = right; L = left; IFG = inferior frontal gyrus; IPL= inferior parietal lobe; ACC: anterior cingulate cortex

**A. Negative Decrease: HC vs BD**

***Positive Negative***

| ***Anatomical*** |  |  |  |  |  |  | ***Anatomical*** |  |  |  |  |  |  |
| --- | --- | --- | --- | --- | --- | --- | --- | --- | --- | --- | --- | --- | --- |
| ***Location*** | ***L/R*** | ***x*** | ***y*** | ***z*** | ***Weights*** | ***KE*** | ***Location*** | ***L/R*** | ***x*** | ***y*** | ***z*** | ***Weights*** | ***KE*** |
| **Insula** | **R** | **44** | **6** | **-12** | **0.0062** | **216** | **Subgenual ACC/caudate** | **L** | **0** | **18** | **-4** | **-0.0065** | **66** |
| **Putamen** | **R** | **26** | **8** | **0** | **0.0049** | **168** | **Cuneus** | **R** | **14** | **-76** | **40** | **-0.0086** | **64** |
| **Mid occipital** | **L** | **-42** | **-78** | **26** | **0.0071** | **143** | **Sup Parietal** | **L** | **-34** | **-68** | **58** | **-0.0083** | **23** |
| **IPL** | **R** | **36** | **-40** | **58** | **0.0059** | **53** | **Precuneus** | **R** | **2** | **-50** | **20** | **-0.0072** | **21** |
| **Supramarginal** | **L** | **-54** | **-46** | **24** | **0.0050** | **49** | **IFG** | **R** | **50** | **48** | **4** | **-0.0064** | **19** |
| **Sup Frontal** | **L** | **-26** | **38** | **34** | **0.0046** | **44** | **Mid Temporal** | **L** | **-62** | **-54** | **14** | **-0.0058** | **18** |
| **IPL** | **R** | **36** | **-66** | **42** | **0.0068** | **41** | **Calcarine** | **L** | **-6** | **-88** | **-12** | **-0.0180** | **18** |
| **IPL** | **L** | **-36** | **-44** | **48** | **0.0051** | **31** | **IFG** | **R** | **48** | **30** | **10** | **-0.0053** | **17** |
| **Cingulate** | **L** | **0** | **10** | **38** | **0.0049** | **27** | **Sup Medial** | **R** | **6** | **28** | **50** | **-0.0060** | **17** |
| **Lingual** | **L** | **-10** | **-74** | **10** | **0.0069** | **24** | **Parahippocampal** | **R** | **22** | **-20** | **-18** | **-0.0062** | **14** |
| **Sup Temporal** | **R** | **38** | **12** | **-30** | **0.0075** | **23** | **IFG** | **R** | **20** | **22** | **-22** | **-0.0057** | **10** |
| **IFG** | **R** | **50** | **14** | **14** | **0.0061** | **22** | **Fusiform** | **R** | **36** | **-34** | **-26** | **-0.0069** | **9** |
| **Precentral** | **L** | **-42** | **-6** | **60** | **0.0062** | **19** | **Supramarginal** | **R** | **62** | **-30** | **36** | **-0.0055** | **7** |
| **Temporal Pole** | **L** | **-52** | **8** | **-8** | **0.0060** | **18** | **Parahippocampal** | **R** | **18** | **-14** | **-20** | **-0.0074** | **7** |
| **ACC** | **L** | **0** | **28** | **20** | **0.0047** | **18** | **Postcentral** | **R** | **14** | **-44** | **80** | **-0.0073** | **5** |
| **Sup frontal** | **R** | **18** | **14** | **70** | **0.0063** | **17** | **Postcentral** | **R** | **14** | **-48** | **78** | **-0.0083** | **5** |
| **Olfactory** | **R** | **26** | **8** | **-18** | **0.0052** | **17** |  |  |  |  |  |  |  |
| **Precuneus** | **L** | **-12** | **-76** | **54** | **0.0110** | **13** |  |  |  |  |  |  |  |
| **Sup occipital** | **L** | **-18** | **-86** | **46** | **0.0110** | **12** |  |  |  |  |  |  |  |
| **Calcarine** | **L** | **-10** | **-84** | **8** | **0.0108** | **7** |  |  |  |  |  |  |  |
| **Inf Temporal** | **R** | **46** | **-50** | **-10** | **0.0066** | **6** |  |  |  |  |  |  |  |
| **Precuneus** | **L** | **-4** | **-42** | **72** | **0.0064** | **6** |  |  |  |  |  |  |  |
| **Precuneus** | **L** | **-16** | **-86** | **12** | **0.0059** | **6** |  |  |  |  |  |  |  |

**B. Neutral Decrease: HC vs BD**

***Positive Negative***

| ***Anatomical*** |  |  |  |  |  |  | ***Anatomical*** |  |  |  |  |  |  |
| --- | --- | --- | --- | --- | --- | --- | --- | --- | --- | --- | --- | --- | --- |
| ***Location*** | ***L/R*** | ***x*** | ***y*** | ***z*** | ***Weights*** | ***KE*** | ***Location*** | ***L/R*** | ***x*** | ***y*** | ***z*** | ***Weights*** | ***KE*** |
| **Mid occipital** | **L** | **-40** | **-82** | **24** | **0.0075** | **165** | **Sup Medial** | **R** | **2** | **34** | **46** | **-0.0059** | **148** |
| **Postcentral/supramarginal** | **R** | **60** | **-16** | **18** | **0.0068** | **87** | **Insula** | **R** | **36** | **24** | **-2** | **-0.0058** | **61** |
| **Mid Frontal** | **L** | **-32** | **40** | **24** | **0.0045** | **67** | **Supramarginal** | **R** | **60** | **-28** | **40** | **-0.0061** | **58** |
| **Sup Frontal** | **L** | **-2** | **32** | **64** | **0.0100** | **58** | **Mid Frontal** | **L** | **-26** | **26** | **52** | **-0.0065** | **56** |
| **Sup Temporal** | **L** | **-56** | **-30** | **18** | **0.0059** | **47** | **Postcentral** | **L** | **-56** | **-14** | **48** | **-0.0074** | **50** |
| **Precentral** | **L** | **-34** | **-8** | **58** | **0.0046** | **42** | **Supramarginal** | **R** | **60** | **-52** | **30** | **-0.0063** | **45** |
| **Inf Temporal** | **L** | **-34** | **8** | **-38** | **0.0054** | **35** | **Cuneus** | **R** | **16** | **-78** | **40** | **-0.0078** | **23** |
| **Mid occipital** | **R** | **34** | **-74** | **18** | **0.0062** | **33** | **Precuneus** | **L** | **-2** | **-46** | **46** | **-0.0056** | **17** |
| **Calcarine** | **L** | **-10** | **-78** | **10** | **0.0078** | **27** | **Sup Temporal** | **R** | **64** | **-8** | **-2** | **-0.0062** | **17** |
| **Sup Temporal** | **L** | **-50** | **-38** | **18** | **0.0055** | **25** | **Supramarginal** | **R** | **52** | **-46** | **36** | **-0.0054** | **16** |
| **Mid Temporal** | **L** | **-58** | **-40** | **-10** | **0.0051** | **24** | **Mid Temporal** | **L** | **-60** | **-46** | **6** | **-0.0060** | **11** |
| **Mid occipital** | **L** | **-28** | **-90** | **24** | **0.0092** | **19** | **Mid Frontal** | **R** | **48** | **50** | **14** | **-0.0065** | **11** |
| **Sup Temporal** | **R** | **66** | **-30** | **16** | **0.0061** | **18** | **ACC** | **R** | **6** | **36** | **-18** | **-0.0057** | **10** |
| **Fusiform** | **L** | **-44** | **-8** | **-24** | **0.0057** | **16** | **Paracentral** | **R** | **4** | **-22** | **80** | **-0.0102** | **10** |
| **Sup Frontal** | **R** | **4** | **56** | **48** | **0.0075** | **15** | **IPL** | **L** | **-56** | **-56** | **36** | **-0.0054** | **9** |
| **Sup Parietal** | **R** | **18** | **-64** | **58** | **0.0072** | **14** | **Sup Frontal** | **R** | **14** | **60** | **26** | **-0.0057** | **9** |
| **Mid Temporal** | **R** | **54** | **-68** | **22** | **0.0068** | **12** | **Postcentral** | **R** | **12** | **-52** | **76** | **-0.0092** | **8** |
| **IFG** | **R** | **20** | **10** | **-24** | **0.0058** | **10** | **Sup Parietal** | **L** | **-36** | **-66** | **58** | **-0.0073** | **7** |
| **Mid Frontal** | **R** | **48** | **12** | **54** | **0.0070** | **9** | **Med Frontal** | **R** | **6** | **52** | **-20** | **-0.0057** | **6** |
| **Postcentral** | **L** | **-18** | **-40** | **66** | **0.0045** | **9** | **Precuneus** | **L** | **0** | **-60** | **48** | **-0.0055** | **5** |
| **Sup occipital** | **R** | **36** | **-76** | **44** | **0.0089** | **7** | **Temporal Pole** | **R** | **54** | **6** | **-2** | **-0.0060** | **5** |
| **Sup Frontal** | **L** | **-16** | **50** | **48** | **0.0059** | **7** |  |  |  |  |  |  |  |
| **Sup occipital** | **L** | **-16** | **-86** | **44** | **0.0119** | **6** |  |  |  |  |  |  |  |
| **Sup Frontal** | **R** | **18** | **70** | **20** | **0.0062** | **6** |  |  |  |  |  |  |  |
| **Mid Frontal** | **R** | **42** | **-6** | **58** | **0.0051** | **6** |  |  |  |  |  |  |  |
| **Sup Frontal** | **R** | **20** | **52** | **48** | **0.0072** | **5** |  |  |  |  |  |  |  |
| **Mid Frontal** | **R** | **32** | **32** | **48** | **0.0046** | **5** |  |  |  |  |  |  |  |

**C. HC: Negative Decrease vs Negative Look**

***Positive Negative***

| ***Anatomical*** |  |  |  |  |  |  | ***Anatomical*** |  |  |  |  |  |  |
| --- | --- | --- | --- | --- | --- | --- | --- | --- | --- | --- | --- | --- | --- |
| ***Location*** | ***L/R*** | ***x*** | ***y*** | ***z*** | ***Weights*** | ***KE*** | ***Location*** | ***L/R*** | ***x*** | ***y*** | ***z*** | ***Weights*** | ***KE*** |
| **Insula** | **R** | **42** | **8** | **-12** | **0.0063** | **70** | **ACC** | **L** | **-4** | **22** | **-10** | **-0.0076** | **150** |
| **IFG** | **L** | **-48** | **42** | **-6** | **0.0076** | **64** | **Fusiform** | **L** | **-50** | **-62** | **-10** | **-0.0059** | **48** |
| **Mid occipital** | **L** | **-40** | **-74** | **4** | **0.0056** | **62** | **Med Frontal** | **L** | **0** | **48** | **-20** | **-0.0063** | **48** |
| **Med Frontal** | **R** | **4** | **58** | **14** | **0.0065** | **60** | **Sup Temporal** | **L** | **-40** | **8** | **-34** | **-0.0061** | **46** |
| **Sup Frontal** | **L** | **-24** | **32** | **48** | **0.0048** | **39** | **Med Frontal** | **L** | **-4** | **56** | **30** | **-0.0072** | **37** |
| **Inf occipital** | **L** | **-26** | **-92** | **-10** | **0.0071** | **38** | **Insula** | **L** | **-40** | **-12** | **22** | **-0.0057** | **34** |
| **Mid occipital** | **R** | **38** | **-74** | **14** | **0.0052** | **29** | **Precentral** | **L** | **-32** | **-6** | **68** | **-0.0074** | **26** |
| **Paracentral** | **L** | **-8** | **-28** | **82** | **0.0091** | **28** | **Mid Frontal** | **L** | **-34** | **36** | **20** | **-0.0053** | **21** |
| **Lentiform Nucleus** | **L** | **-20** | **16** | **2** | **0.0051** | **22** | **IFG** | **R** | **24** | **10** | **-30** | **-0.0078** | **18** |
| **Mid Temporal** | **L** | **-48** | **-74** | **16** | **0.0055** | **19** | **Med Frontal** | **R** | **12** | **70** | **4** | **-0.0109** | **13** |
| **Mid Temporal** | **R** | **58** | **-62** | **6** | **0.0065** | **18** | **IPL** | **L** | **-50** | **-26** | **52** | **-0.0053** | **9** |
| **Mid occipital** | **R** | **28** | **-88** | **-8** | **0.0059** | **17** | **Uncus** | **R** | **26** | **8** | **-38** | **-0.0058** | **8** |
| **Mid Temporal** | **L** | **-40** | **-64** | **34** | **0.0050** | **17** | **Precuneus** | **R** | **14** | **-60** | **32** | **-0.0053** | **7** |
| **Lingual** | **L** | **-12** | **-90** | **8** | **0.0056** | **15** | **Cingulate** | **L** | **-2** | **-10** | **36** | **-0.0070** | **7** |
| **Inf Parietal** | **R** | **42** | **-60** | **44** | **0.0048** | **13** | **Sup Frontal** | **L** | **-4** | **70** | **14** | **-0.0068** | **6** |
| **IFG** | **R** | **34** | **18** | **-22** | **0.0073** | **12** | **Sup Temporal** | **L** | **-64** | **-44** | **24** | **-0.0054** | **5** |
| **Sup Frontal** | **L** | **-22** | **44** | **42** | **0.0051** | **12** | **Cingulate** | **R** | **6** | **-40** | **28** | **-0.0054** | **5** |
| **Mid Frontal** | **R** | **36** | **40** | **34** | **0.0059** | **6** | **Precentral** | **L** | **-12** | **-26** | **72** | **-0.0057** | **5** |
| **Postcentral** | **R** | **68** | **-8** | **12** | **0.0047** | **6** | **Med Frontal** | **R** | **16** | **62** | **-4** | **-0.0103** | **5** |
| **Precentral** | **L** | **-64** | **2** | **24** | **0.0044** | **5** |  |  |  |  |  |  |  |
|  |  |  |  |  |  |  |  |  |  |  |  |  |  |

**D. HC: Neutral Decrease vs Neutral Look**

***Positive Negative***

| ***Anatomical*** |  |  |  |  |  |  | ***Anatomical*** |  |  |  |  |  |  |
| --- | --- | --- | --- | --- | --- | --- | --- | --- | --- | --- | --- | --- | --- |
| ***Location*** | ***L/R*** | ***x*** | ***y*** | ***z*** | ***Weights*** | ***KE*** | ***Location*** | ***L/R*** | ***x*** | ***y*** | ***z*** | ***Weights*** | ***KE*** |
| **Caudate** | **R** | **14** | **14** | **-6** | **0.0065** | **126** | **Sup Parietal** | **R** | **32** | **-52** | **48** | **-0.0062** | **81** |
| **Med Frontal** | **L** | **0** | **6** | **56** | **0.0069** | **116** | **IPL** | **L** | **-34** | **-46** | **44** | **-0.0060** | **74** |
| **Precuneus** | **R** | **10** | **-72** | **30** | **0.0057** | **66** | **Lingual** | **R** | **4** | **-80** | **4** | **-0.0086** | **51** |
| **Mid Temporal** | **R** | **54** | **-56** | **2** | **0.0063** | **60** | **Precuneus** | **R** | **26** | **-68** | **46** | **-0.0060** | **34** |
| **Mid Frontal** | **L** | **-24** | **44** | **-12** | **0.0054** | **58** | **Mid Frontal** | **L** | **-28** | **-2** | **66** | **-0.0058** | **22** |
| **Postcentral** | **R** | **50** | **-26** | **52** | **0.0052** | **55** | **Sup Frontal** | **L** | **-6** | **66** | **20** | **-0.0090** | **18** |
| **Mid occipital** | **R** | **42** | **-80** | **8** | **0.0104** | **51** | **Mid occipital** | **R** | **32** | **-92** | **16** | **-0.0058** | **17** |
| **Sup Temporal** | **R** | **66** | **-28** | **16** | **0.0064** | **40** | **Fusiform** | **R** | **42** | **-82** | **-10** | **-0.0073** | **16** |
| **Precentral** | **L** | **-44** | **24** | **36** | **0.0059** | **31** | **Sup Frontal** | **R** | **44** | **54** | **14** | **-0.0060** | **13** |
| **Lingual** | **R** | **28** | **-60** | **8** | **0.0055** | **30** | **Precentral** | **L** | **-60** | **6** | **30** | **-0.0056** | **12** |
| **Sup Temporal** | **L** | **-54** | **-44** | **22** | **0.0058** | **29** | **Sup Temporal** | **L** | **-44** | **6** | **-16** | **-0.0074** | **12** |
| **Sup Frontal** | **L** | **-36** | **58** | **14** | **0.0082** | **19** | **Mid Frontal** | **R** | **36** | **14** | **62** | **-0.0082** | **10** |
| **Precuneus** | **L** | **-14** | **-70** | **62** | **0.0075** | **18** | **IFG** | **L** | **-30** | **18** | **-20** | **-0.0054** | **8** |
| **Lentiform Nucleus** | **L** | **-18** | **16** | **-4** | **0.0053** | **9** | **Postcentral** | **R** | **12** | **-54** | **78** | **-0.0066** | **8** |
| **Cingulate** | **L** | **-14** | **-20** | **46** | **0.0045** | **8** | **Sup Temporal** | **R** | **58** | **8** | **-12** | **-0.0059** | **6** |
| **Sup Frontal** | **R** | **34** | **32** | **48** | **0.0064** | **6** | **Sup Frontal** | **R** | **32** | **-12** | **72** | **-0.0069** | **5** |
| **IFG** | **R** | **56** | **32** | **6** | **0.0050** | **6** | **Precuneus** | **L** | **0** | **-58** | **54** | **-0.0074** | **5** |
| **Sup Frontal** | **L** | **-18** | **14** | **72** | **0.0066** | **5** | **Sup Frontal** | **R** | **2** | **68** | **22** | **-0.0093** | **5** |
|  |  |  |  |  |  |  |  |  |  |  |  |  |  |

**E. BD: Negative Decrease vs Negative Look**

***Positive Negative***

| ***Anatomical*** |  |  |  |  |  |  | ***Anatomical*** |  |  |  |  |  |  |
| --- | --- | --- | --- | --- | --- | --- | --- | --- | --- | --- | --- | --- | --- |
| ***Location*** | ***L/R*** | ***x*** | ***y*** | ***z*** | ***Weights*** | ***KE*** | ***Location*** | ***L/R*** | ***x*** | ***y*** | ***z*** | ***Weights*** | ***KE*** |
| **Lingual** | **L** | **-4** | **-78** | **8** | **0.0083** | **109** | **IFG** | **R** | **42** | **50** | **-6** | **-0.0079** | **306** |
| **Sup Frontal** | **L** | **-6** | **58** | **38** | **0.0061** | **79** | **Insula** | **R** | **42** | **4** | **-10** | **-0.0079** | **70** |
| **Post Cingulate** | **L** | **-4** | **-44** | **26** | **0.0056** | **72** | **Lingual** | **L** | **-8** | **-98** | **2** | **-0.0082** | **38** |
| **Insula** | **L** | **-34** | **22** | **-6** | **0.0049** | **48** | **Precuneus** | **R** | **40** | **-64** | **40** | **-0.0059** | **32** |
| **ACC** | **L** | **-6** | **26** | **-6** | **0.0064** | **23** | **Lingual** | **R** | **14** | **-54** | **-4** | **-0.0092** | **32** |
| **IFG** | **L** | **-54** | **28** | **12** | **0.0050** | **17** | **Precuneus** | **L** | **-8** | **-48** | **42** | **-0.0058** | **25** |
| **IPL** | **R** | **52** | **-36** | **44** | **0.0044** | **17** | **Sup Frontal** | **R** | **28** | **58** | **26** | **-0.0062** | **22** |
| **Posterior Mid Frontal** | **R** | **4** | **-10** | **76** | **0.0087** | **13** | **Parahippocampus** | **R** | **32** | **-44** | **-8** | **-0.0073** | **22** |
| **Sup Frontal** | **R** | **22** | **26** | **62** | **0.0049** | **11** | **Insula** | **L** | **-42** | **-28** | **14** | **-0.0063** | **11** |
| **Mid occipital** | **L** | **-42** | **-84** | **8** | **0.0047** | **11** | **Mid Frontal** | **L** | **-50** | **20** | **32** | **-0.0061** | **9** |
| **Mid Temporal** | **R** | **66** | **-46** | **0** | **0.0050** | **10** | **Caudate** | **L** | **-16** | **20** | **8** | **-0.0067** | **9** |
| **Mid Temporal** | **L** | **-60** | **4** | **-22** | **0.0051** | **8** | **Sup Frontal** | **L** | **-20** | **38** | **48** | **-0.0056** | **8** |
| **Mid Temporal** | **R** | **58** | **-58** | **-8** | **0.0048** | **8** | **Mid Frontal** | **L** | **-50** | **34** | **28** | **-0.0061** | **5** |
| **Mid Frontal** | **R** | **52** | **32** | **12** | **0.0046** | **8** |  |  |  |  |  |  |  |
| **Mid Temporal** | **L** | **-56** | **8** | **-26** | **0.0051** | **7** |  |  |  |  |  |  |  |
| **Sup Temporal** | **R** | **60** | **-54** | **28** | **0.0052** | **5** |  |  |  |  |  |  |  |
| **Precuneus** | **R** | **28** | **-80** | **40** | **0.0047** | **5** |  |  |  |  |  |  |  |
| **Precuneus** | **L** | **-26** | **-76** | **36** | **0.0042** | **5** |  |  |  |  |  |  |  |

**F. BD: Neutral Decrease vs Neutral Look**

***Positive Negative***

| ***Anatomical*** |  |  |  |  |  |  | ***Anatomical*** |  |  |  |  |  |  |
| --- | --- | --- | --- | --- | --- | --- | --- | --- | --- | --- | --- | --- | --- |
| ***Location*** | ***L/R*** | ***x*** | ***y*** | ***z*** | ***Weights*** | ***KE*** | ***Location*** | ***L/R*** | ***x*** | ***y*** | ***z*** | ***Weights*** | ***KE*** |
| **Mid Temporal** | **R** | **64** | **-28** | **-14** | **0.0057** | **93** | **Med Frontal** | **L** | **-8** | **50** | **-18** | **-0.00759** | **50** |
| **Cingulate** | **L** | **-4** | **12** | **42** | **0.00525** | **52** | **Precentral** | **L** | **-48** | **-10** | **54** | **-0.00583** | **39** |
| **Precentral** | **L** | **-60** | **6** | **12** | **0.00592** | **38** | **Sup Frontal** | **R** | **6** | **64** | **16** | **-0.00952** | **35** |
| **IFG** | **L** | **-26** | **22** | **-20** | **0.00522** | **37** | **Caudate** | **R** | **16** | **20** | **10** | **-0.00611** | **33** |
| **Mid Temporal** | **R** | **42** | **-10** | **-36** | **0.00489** | **29** | **Precentral** | **L** | **-22** | **-12** | **74** | **-0.0079** | **19** |
| **Mid Frontal** | **R** | **36** | **58** | **-6** | **0.00781** | **23** | **Precentral** | **R** | **14** | **-32** | **84** | **-0.00974** | **17** |
| **Mid Frontal** | **R** | **40** | **18** | **56** | **0.00633** | **22** | **Mid Frontal** | **R** | **52** | **36** | **24** | **-0.0059** | **14** |
| **Precentral** | **R** | **50** | **26** | **36** | **0.00825** | **21** | **Med Frontal** | **L** | **-4** | **60** | **-16** | **-0.00718** | **12** |
| **Sup Frontal** | **L** | **-16** | **58** | **12** | **0.00537** | **18** | **Sup Frontal** | **R** | **14** | **8** | **76** | **-0.00858** | **12** |
| **Anterior Cingulate** | **L** | **0** | **36** | **18** | **0.00554** | **17** | **Subcallosal** | **R** | **18** | **2** | **-16** | **-0.0065** | **7** |
| **Sup Frontal** | **R** | **20** | **62** | **8** | **0.00569** | **16** | **Precentral** | **R** | **46** | **-6** | **56** | **-0.00574** | **6** |
| **Precuneus** | **L** | **-10** | **-44** | **34** | **0.00473** | **16** | **Thalamus** | **L** | **-4** | **-20** | **4** | **-0.00582** | **6** |
| **Parahippocampus** | **R** | **24** | **-16** | **-26** | **0.00555** | **13** | **Sup Temporal** | **L** | **-60** | **0** | **-6** | **-0.00607** | **6** |
| **Uncus** | **L** | **-20** | **-2** | **-32** | **0.00767** | **9** | **Mid occipital** | **L** | **-22** | **-100** | **8** | **-0.00666** | **6** |
| **Angular** | **L** | **-46** | **-64** | **40** | **0.00563** | **9** | **Parahippocampus** | **L** | **-28** | **-50** | **0** | **-0.00589** | **5** |
| **Sup Parietal** | **L** | **-22** | **-60** | **60** | **0.00514** | **9** | **Mid Frontal** | **R** | **24** | **34** | **-22** | **-0.00772** | **5** |
| **Mid Temporal** | **L** | **-64** | **-40** | **-16** | **0.00814** | **8** |  |  |  |  |  |  |  |
| **Mid Frontal** | **L** | **-42** | **36** | **8** | **0.00449** | **8** |  |  |  |  |  |  |  |
| **Inf Temporal** | **R** | **60** | **-58** | **-12** | **0.00586** | **7** |  |  |  |  |  |  |  |
| **IFG** | **R** | **52** | **38** | **-2** | **0.00526** | **7** |  |  |  |  |  |  |  |
| **IFG** | **L** | **-42** | **34** | **4** | **0.00457** | **6** |  |  |  |  |  |  |  |
| **Uncus** | **L** | **-28** | **-12** | **-32** | **0.00666** | **5** |  |  |  |  |  |  |  |
| **Parahippocampus** | **L** | **-14** | **-6** | **-16** | **0.00656** | **5** |  |  |  |  |  |  |  |
| **Mid Temporal** | **R** | **66** | **-12** | **-14** | **0.00464** | **5** |  |  |  |  |  |  |  |

**Table S4: Top 10 regions contributing to the significant group classifications based on the combined cluster size**

|  |  | **Negative Decrease HC vs BD** | |  |  |  | **Neutral Decrease HC vs BD** | |  |
| --- | --- | --- | --- | --- | --- | --- | --- | --- | --- |
|  | ***Positive*** | | ***Negative*** | |  | ***Positive*** | | ***Negative*** | |
| 1 | **Insula** | | **Subgenual ACC/caudate** | | 1 | **Mid occipital** | | **Sup Medial** | |
| 2 | **Putamen** | | **Cuneus** | | 2 | **Postcentral/supramarginal** | | **IPL** | |
| 3 | **Mid occipital** | | **IFG** | | 3 | **Sup Frontal** | | **Mid Frontal** | |
| 4 | **IPL** | | **Sup Parietal** | | 4 | **IPL** | | **Insula** | |
| 5 | **Sup Frontal** | | **precuneus** | | 5 | **Mid Frontal** | | **Postcentral** | |
| 6 | **Angular** | | **Parahippocampus** | | 6 | **Precentral** | | **Cuneus** | |
| 7 | **Cingulate** | | **Mid Temporal** | | 7 | **Mid Temporal** | | **Precuneus** | |
| 8 | **Precuneus** | | **Postcentral** | | 8 | **Inf Temporal** | | **Sup Temporal** | |
| 9 | **Lingual** | | **Calcarine** | | 9 | **Calcarine** | | **Mid Temporal** | |
| 10 | **Sup Temporal** | | **Sup Medial** | | 10 | **Fusiform** | | **ACC** | |

Anatomically defined clusters corresponding to the top 5% of the weight contribution in significant group classifications (HC vs BD) were combined based on the MNI anatomical location.

**Supplementary Figures**

**Figure S1:**  **Statistical parametric maps of the contrast *Negative Decrease – Negative Look* and *Neutral Decrease – Neutral Look*.**


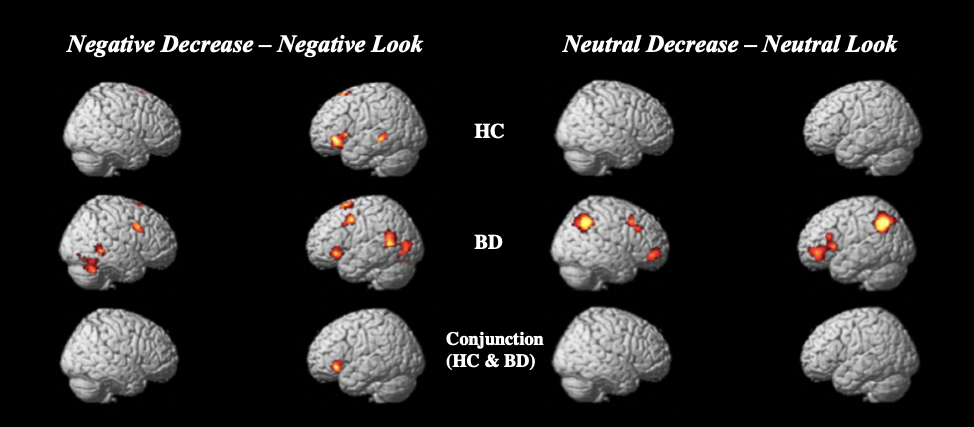


Rendering of the significant activations for the Decrease minus Look contrast for Negative (Left) and Neutral (Right) images, for HC (Top), BD (Middle), and the conjunction of the two groups (Bottom). Coordinates and z-scores are shown in Table S2.

**Figure S2:** **Weight map of positive and negative voxel-wise weight contribution in the within-group classifications**

**A. HC: *Negative Decrease vs Negative Look*, B. HC: *Neutral Decrease vs Neutral Look*, C. BD: *Negative Decrease vs Negative Look*, D. BD: *Neutral Decrease vs Neutral Look***

Highlighted region depicts contribution of the top 5% positive (red) and negative (blue) weights, with a minimum extent of 5 voxels. Abbreviations are as follows: a.u. = arbitrary units;

Mid OCC= middle occipital cortex; IFG = inferior frontal gyrus; IPL = inferior parietal lobe; ACC = anterior cingulate cortex; Med frontal = medial frontal; Sup Frontal = superior frontal; PCC = posterior cingulate cortex; Mid Temporal = middle temporal; Sup Parietal = superior parietal. Images are displayed in neurological convention (i.e., right hemisphere is shown on the right)

**Figure S3:** **Amygdala activation for the contrast *Negative Look* – *Negative Decrease* for the HC group (x = 20, y = -4, z = -18).**


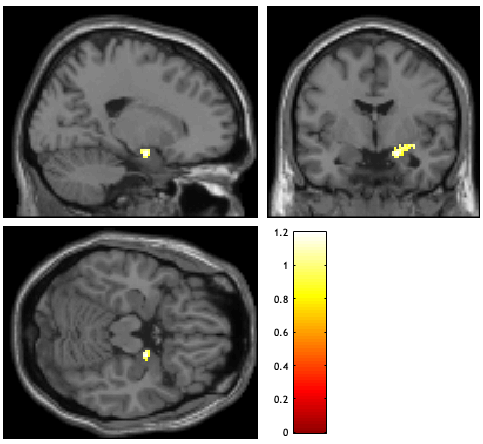


Images are displayed in neurological convention (i.e., right hemisphere is shown on the right)
